# Bovine natural killer cell subsets can be defined by differential expression of CD161 (*KLRB1*)

**DOI:** 10.3389/fimmu.2026.1843038

**Published:** 2026-06-03

**Authors:** Inga Dry, Marta Kalandadze, Zhiguang Wu, Irene C. McGuinnes, Matthew D. Edmans, Lindert Benedictus, Timothy Connelley, Jayne C. Hope, Lindsey A. Waddell

**Affiliations:** 1 The Roslin Institute and R(D)SVS, University of Edinburgh, Edinburgh, United Kingdom; 2 Peter Medawar Building for Pathogen Research, Nuffield Department of Medicine, University of Oxford, Oxford, United Kingdom; 3 Division of Farm Animal Health, Faculty of Veterinary Medicine, Utrecht University, Utrecht, Netherlands

**Keywords:** cattle, CD161, immune, KLRB1, natural killer cells

## Abstract

Natural killer (NK) cells play a vital role in the immune response to infection, disease, and vaccination. Bovine NK cell subsets have been described; however, a more in-depth characterization is currently hindered by the lack of species-specific reagents in comparison to humans and mice. CD161, encoded by the *KLRB1* gene, is a C-type lectin-like receptor known to be highly expressed in human NK cells. CD161 has been described in human immunity as defining a pro-inflammatory subset of NK cells that are capable of responding to cytokines. The specific function and importance of bovine CD161^+^ cells have yet to be defined. This study developed and characterized a novel mouse anti-bovine CD161 monoclonal antibody and determined the expression of CD161 on NK cells, CD8 T cells, and gamma delta T (γδT) cells in peripheral blood from calves. Future research will investigate the specific function and targeting of the described CD161^+^ subsets in baseline immune response, infection, and response to vaccination.

## 1 Introduction

CD161 is a C-type lectin-like receptor expressed on the cell surface of the majority of natural killer (NK) cells and specific subsets of T cells in humans (1). Expression has been described during NK cell development (2), with staining in umbilical cord blood in infants suggestive of the presence of CD161 within the early stages of development (1). In human peripheral blood, CD161 expression has been reported on one CD4^+^CD161^+^, and two CD8^+^CD161^+^ T-cell subsets (3). T-cell subsets that express CD161 have been shown to be responsive to cytokines such as interleukin 12 (IL-12) and IL-18, and these cell types have therefore been described as possessing an innate-like function (4). Notably, types of human T cells that express CD161 are gamma delta T (γδT) cells (5) and mucosal-associated invariant T (MAIT) cells (6). CD161^+^ NK cells are innate cells that are responsive to IL-12 and IL-18 (3). CD161 expression has been described on a pro-inflammatory subset of human NK cells, and binding to its ligand CLEC2D (LLT1) can inhibit cytotoxicity and cytokine production (1). The pro-inflammatory nature of CD161^+^ NK cells in humans suggests roles in the control of infection and the regulation of disease processes. Human NK cell subsets are routinely defined by the expression of CD56; however, this gene is absent in cattle (7). Bovine NK cell subsets with divergent functions are identified by the expression of NKp46 (*NCR1*, CD335) and CD2, and the absence of CD3 (8). The CD2^−^NKp46^+^ subset is the main cytokine-producing subset, predominantly producing interferon gamma (IFNγ) (9). This IFNγ response is central to the vaccination response to Bacille Calmette–Guerin (BCG) in calves and in neonatal humans, underpinning protective immunity to tuberculosis (TB).

Cattle and humans each encode only one *KLRB1* gene, in contrast to rodents that encode multiple genes (10). Therefore, findings from human CD161 biology could potentially apply to cattle, and if such is the case, cattle could be a suitable model to study the role of CD161 in human disease and infection. However, studies into the biology of CD161 in cattle have been hindered by the lack of reagents specifically recognizing bovine CD161.

Here, we describe the generation of a novel antibody specific for bovine CD161 and confirm the expression of CD161 by subsets of NK, CD8^+^ T, and γδTCR^+^ T cells within the peripheral blood in cattle. This monoclonal antibody (mAb) will enable further studies on the role of CD161 in the immune responses of cattle.

## 2 Materials and methods

All experimental protocols were carried out under the authority of a UK Home Office Project Licence under the regulations of the Animals (Scientific Procedures) Act 1986, with approval from The Roslin Institute’s Local Animal Welfare and Ethical Review Board (AWERB). The results are reported in line with the Animal Research: Reporting of *In Vivo* Experiments (ARRIVE) guidelines (11).

### 2.1 Construction and purification of recombinant bovine CD161-HuIgG1-Fc purified protein

The sequence corresponding to the extracellular domain of bovine CD161 (NCBI reference no. XM0052070515) was synthesized with an *Eco*RV restriction site at the 5′-end and a *Bgl*II restriction enzyme site at the 3′-end (SynBio Technologies, Monmouth Junction, NJ, USA) and cloned into the expression vector pFUSE-hIgG1-Fc2 (InvivoGen, San Diego, CA, USA). The plasmid was sequenced using Sanger sequencing (LightRun Tube Services; Eurofins, Ebersberg, Germany) to confirm the open reading frame (ORF) prior to the initiation of expression work. Recombinant bovine CD161-FC (rBvCD161-FC) was purified from the supernatant of Lipofectamine2000 (Life Technologies, Carlsbad, CA, USA) transfected HEK293T cells maintained in Dulbecco’s modified Eagle’s medium (DMEM) (Merck, Darmstadt, Germany), 10% immunoglobulin G (IgG)-depleted UltraLow fetal bovine serum (FBS) (Gibco, Waltham, MA, USA), and 1× Glutamax (Gibco) media using a HiTrap Protein G HP antibody purification column (Cytiva, Marlborough, MA, USA) according to the manufacturer’s instructions. Purified rBvCD161-FC was dialyzed using a Slide-A-Lyzer™ G3 Dialysis cassette 10 kDa MWCO (ThermoFisher Scientific, Waltham, MA, USA) into phosphate-buffered saline (PBS) and assessed for identity and purity using mass spectrometry by the Proteomics and Metabolomics Facility at the Roslin Institute, University of Edinburgh, prior to use as an immunogen.

### 2.2 Mouse immunization for hybridoma production

Three Balb/c mice (Charles River Laboratories, Margate, UK) were immunized for mAb production. To increase the applications of the mAbs produced, animals were co-immunized with rBvCD161-FC and porcine CD161-HuIgG1-Fc recombinant proteins (12). Initial pre-immunization bleeds were taken from each animal, followed by three subcutaneous injections with 50 µg protein with TiterMax Gold adjuvant (Sigma-Aldrich, St. Louis, MO, USA) at a minimum of 14-day intervals. A final intraperitoneal injection with 50 µg antigen with no adjuvant was performed 3 days prior to tissue harvest and fusion.

### 2.3 Confirmation of response to immunogen by indirect ELISA

Pre- and post-immunization sera from all mice were screened with indirect ELISA to confirm the response to the rBvCD161-FC immunogen. Overnight, 96-well MaxiSorp ELISA plates (Nunc, Roskilde, Denmark) were coated with 50 ng/well of rBvCD161-FC or recombinant HuIgG1-Fc (rHu-FC, produced in-house). The following day, an indirect ELISA was carried out exactly as previously described (13) using mouse serum diluted 1:1,000 in PBS followed by a horse anti-mouse IgG-HRP (7076S; Cell Signaling, Danvers, MA, USA) secondary antibody diluted 1:5,000 in PBS.

### 2.4 Fusion and hybridoma production

Splenocytes were isolated and the fusion of hybridomas carried out in accordance with the method cited by Khalid et al. (14). Indirect ELISA as described above was used to screen neat hybridoma supernatant against the rBvCD161-FC immunogen, and rHu-FC was utilized to determine which polyclonal hybridomas to take forward to mAb production.

### 2.5 Monoclonal antibody production and purification

Following the expansion of selected clones taken forward from ELISA, single-cell sorting was carried out using a BD FACS Aria III (BD Biosciences, San Jose, CA, USA) as previously described (14). Following saturation of the cell culture supernatant, indirect ELISA as above was performed on neat polyclonal hybridoma supernatant or purified mAb diluted in PBS to determine mAb clones specific against the rBvCD161-FC immunogen. The two most strongly reactive mAbs were expanded in culture and purified mAb produced by passing the cell culture supernatant over a HiTrap Protein G column (Cytiva) according to the manufacturer’s instructions. The purified antibody was dialyzed using a Slide-A-Lyzer™ G3 Dialysis cassette 10 kDa MWCO (ThermoFisher Scientific) into PBS, aliquoted, and stored at −20°C until utilized.

### 2.6 PBMC isolation

During a previous study, peripheral blood mononuclear cells (PBMCs) were isolated and cryopreserved from Holstein–Friesian calves as previously described (15). In summary, heparinized blood was collected, diluted 1:1 with PBS, and layered over Lymphoprep Density Gradient (STEMCELL Technologies UK Ltd., Cambridge, UK). Layered blood was centrifuged at 1,200 × *g* for 40 min with no brake. The PBMC cell layer was carefully removed, washed in PBS, and counted using Trypan blue (Gibco) to determine cell viability. Isolated PBMCs were cryopreserved in fetal bovine serum (FBS) (Gibco) containing 10% dimethyl sulfoxide (DMSO) (Sigma-Aldrich) and stored at −155°C for future use.

### 2.7 Flow cytometry

Specific binding of mouse anti-bovine CD161 mAbs to bovine cells was confirmed by flow cytometric analysis of PBMCs. Following recovery from cryopreservation, 1 × 10^6^ cells/sample were blocked in PBS/5% normal goat serum (NGS) for 15 min on ice. Following centrifugation at 400 × *g* for 5 min and removal of the supernatant, 50 µl/well primary mAbs diluted in PBS/5% NGS was added and incubated for 30 min on ice. In the instance of the primary mAbs being unconjugated, three PBS washes were carried out, and following final centrifugation, 50 µl/well of mouse anti-IgG1-FITC secondary antibody (406605; BioLegend, San Diego, CA, USA) diluted 1:200 in PBS was added and incubated on ice for 30 min. A further three PBS washes were carried out and Sytox Blue (Invitrogen, Carlsbad, CA, USA) viability dye diluted in PBS added prior to analysis on a BD LSR Fortessa flow cytometer (BD Biosciences). For multi-parametric flow cytometry, Zombie NIR viability dye (BioLegend) was used and the cells fixed in 2% paraformaldehyde (PFA) prior to storage overnight at 4°C. The following day, UltraComp beads (ThermoFisher Scientific) were used for compensation and approximately 250,000 events within the lymphocyte region of PBMCs (**Supplementary Figure 1**) were collected. Analysis was carried out using FlowJo v10 software (BD Biosciences). Cells only, cells plus viability dye only, secondary antibody only, isotype control, and fluorescence minus one (FMO) controls were included, where required. Example gating strategies are described in **Supplementary Figure 1**. Information on all antibodies used within multi-parametric panels is listed in **Table 1**.

**Table 1:** Primary antibodies used within the multicolor flow cytometry staining panels.

Antigen	Specificity	Clone	Isotype	Fluorophore
CD2	Mouse anti-bovine	CC42	IgG1	AF488
CD8	Mouse anti-bovine	CC63	IgG1	FITC
CD161	Mouse anti-bovine	1F2/3A4	IgG1	AF568
NKp46	Mouse anti-bovine	Gr13.1	IgG1	AF647
γδTCR	Mouse anti-bovine	GB21A	IgG2b	AF647
CD4	Mouse anti-bovine	IL-A12	IgG2a	AF647

When required, in-house conjugation of the primary antibodies with Alexa Fluor dyes was carried out according to the kit manufacturer’s instructions (Molecular Probes, Eugene, OR, USA).

## 3 Results

### 3.1 Response of mice to immunization with recombinant bovine CD161-FC

**Figure 1:**
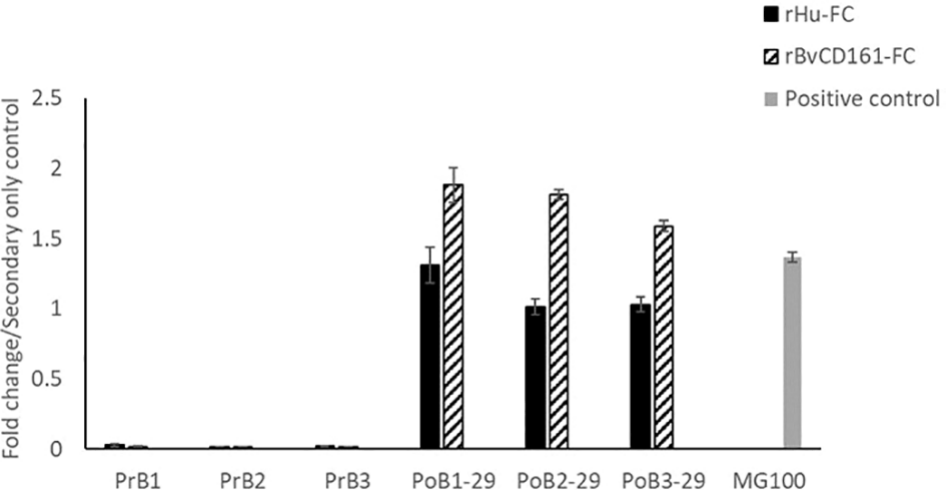
Response of immunized mice by indirect ELISA. A tail bleed from each mouse was taken pre-immunization (*PrB*) at day 0 and post-second immunization (*PoB*) at day 29. An indirect ELISA was carried out against recombinant bovine CD161-FC protein (rBvCD161-FC) used as the immunogen (*striped*) in addition to the recombinant HuIgG1-FC (rHu-FC) tag (*black*). Mouse IgG serum (MG100) was used as a positive control (*gray*).

Indirect ELISAs on the pre- and post-immunization bleeds from immunized mice showed the response of each animal to the rBvCD161-FC recombinant protein antigen in comparison to the rHu-FC irrelevant tag (**Figure 1**).

A high response against the CD161 immunogen was observed in all three mice following the second immunization in comparison to the pre-bleed.

### 3.2 Specificity of CD161 mAb to bovine CD161 by indirect ELISA

**Figure 2:**
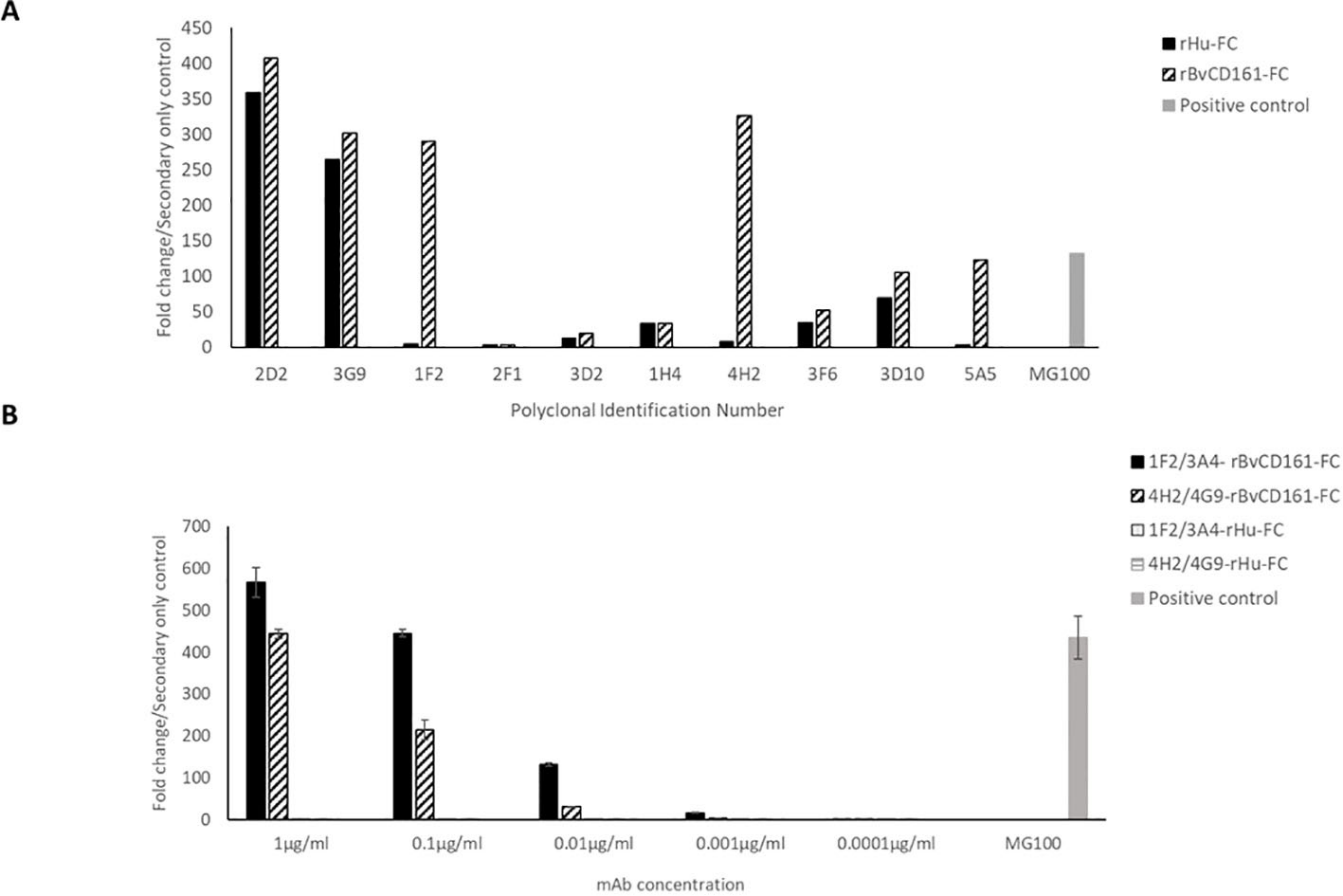
Polyclonal hybridoma and monoclonal antibody (mAb) screening by indirect ELISA. Indirect ELISAs were carried out against recombinant bovine CD161-FC protein (rBvCD161-FC) used as the immunogen (*striped*) and recombinant HuIgG1-FC (rHu-FC) tag (*black*) **(A)**. Following mAb purification, clones 4H2/4G9 and 1F2/3A4 were assessed for response to the rBvCD161-FC protein, and the irrelevant rHu-FC tag at reducing concentrations **(B)**. Mouse IgG serum (MG100) was used as a positive control (*gray*).

Following polyclonal hybridomas reaching saturation in HT media, the cell culture supernatant was screened using indirect ELISA. Polyclonal hybridomas 1F2 and 4H2 showed strong reactivity against the rBvCD161-FC immunogen and a very low response to the irrelevant rHu-FC tag (**Figure 2A**). These two wells were expanded and taken forward for mAb selection by fluorescent-activated cell sorting (FACS).

Following purification, the mAb clones 1F2/3A4 and 4H2/4G9 were further screened using indirect ELISA at reducing concentrations and confirmed the high specificity of both mAbs against the rBvCD161-FC immunogen in comparison to the irrelevant rHu-FC tag (**Figure 2B**).

### 3.3 Specificity of CD161 mAb to bovine PBMCs by flow cytometry

**Figure 3:**
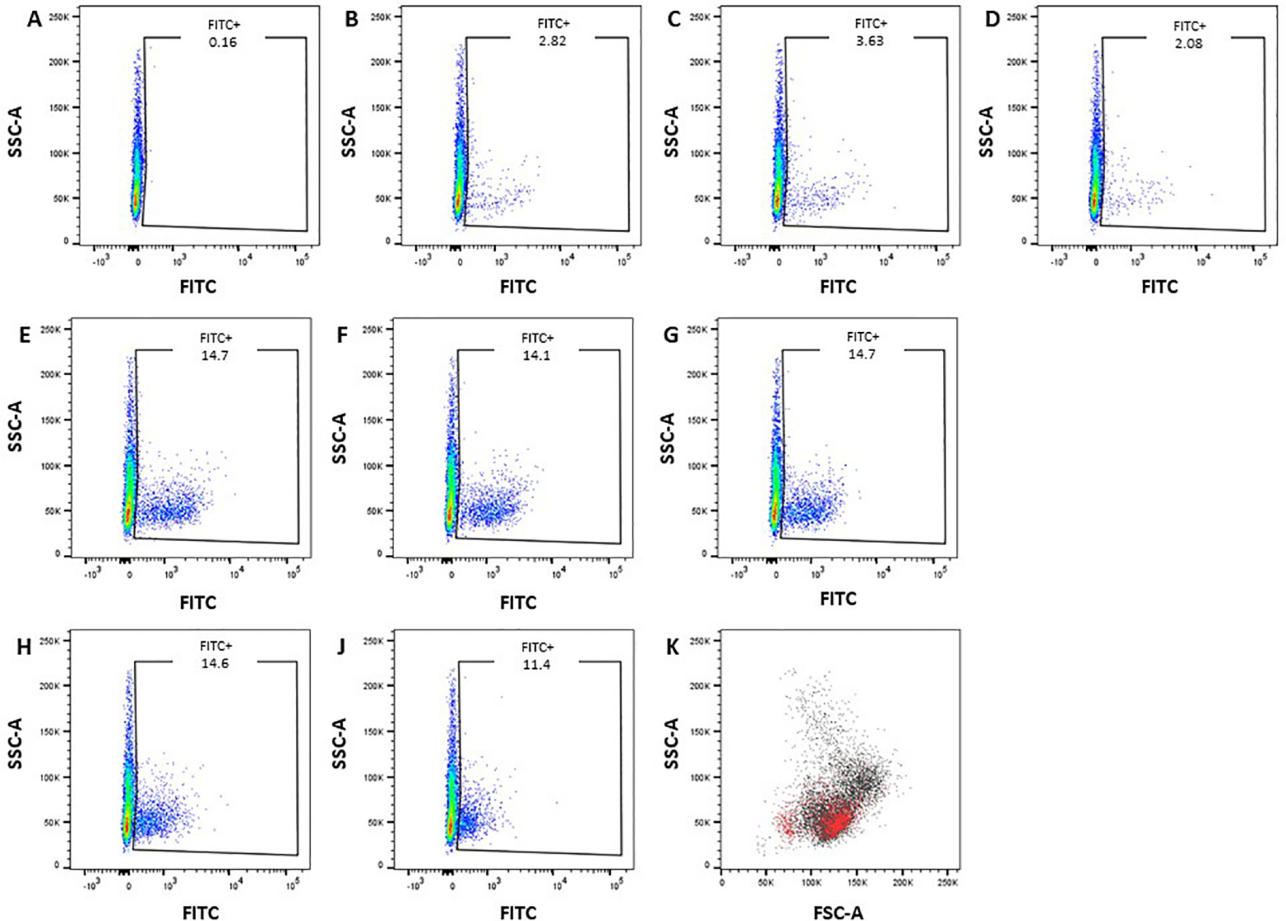
Testing of purified monoclonal antibody (mAb) 1F2/3A4 by flow cytometry against bovine peripheral blood mononuclear cells (PBMCs). The specificity of the purified mouse anti-bovine CD161 mAb clone 1F2/3A4 was confirmed in bovine PBMCs by flow cytometry. **(A–D)** Cells only **(A)**, secondary antibody only **(B)**, and mouse IgG1 isotype control antibody (clone MG1-45) at 4 µg/ml **(C)** and 1 µg/ml **(D)** were included. **(E–J)**. Purified mAb at concentrations decreasing by half from 4 to 0.25 µg/ml were tested. Backgating of the antibody-positive population at 2 µg/ml mAb was performed against the original FSC-A *vs*. SSC-A profile. All plots were gated on single, live cells as described in **Supplementary Figure 1A**.

To confirm specific binding of the anti-CD161 mAb clone 1F2/3A4 to natively expressed CD161, PBMCs were stained with twofold dilutions of purified mAb (4–0.25 µg/ml) followed by an anti-mouse IgG1-FITC secondary antibody (BioLegend) and detection by flow cytometry (**Figures 3E–J**).

Based on clear distinction between positive and negative staining (**Figures 3A–D**), an optimal staining concentration of 2 µg/ml was determined (**Figure 3F**). Backgating of the positive population to the FSC-A *vs*. SSC-A plot of the PBMCs confirmed that the expression of CD161 was predominantly within the lymphocyte region (**Figure 3K**).

### 3.4 CD161 co-expression on NK, γδT, and T cells in peripheral blood

**Figure 4:**
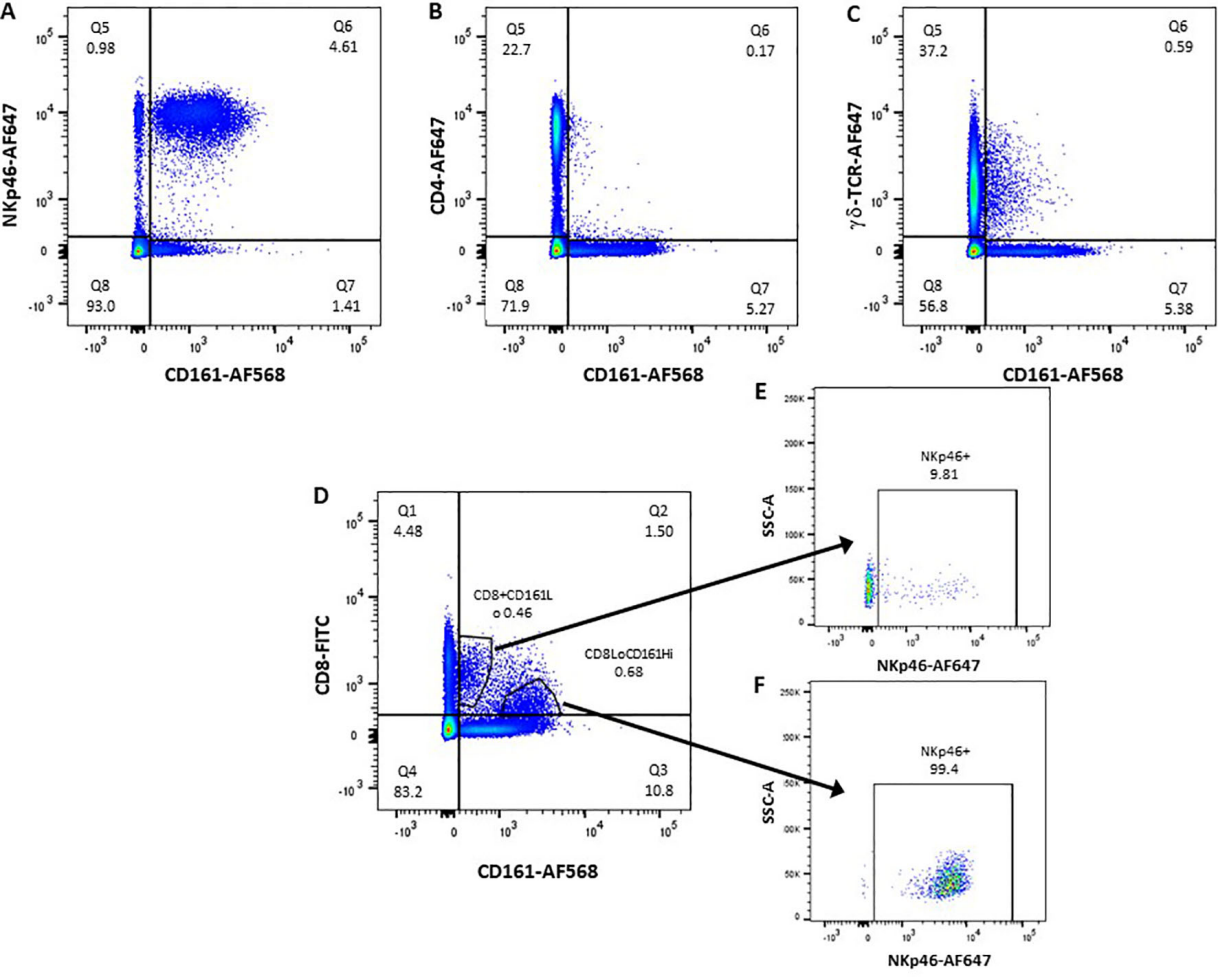
Expression of CD161 on CD4, CD8, and gamma delta (γδ) T cells in bovine peripheral blood. To determine the expression of CD161 on T cells, multicolor flow cytometry was performed. **(A–D)** Co-expression with NKp46 **(A)**, CD4 **(B)**, γδTCR **(C)**, and CD8 **(D)** was measured. **(E, F)** Subsequent NKp46 expression on the CD8/CD161 subsets was also determined. All plots were gated on single, live cells as described in **Supplementary Figure 1B**, and quadrant gates were drawn based on relevant fluorescence minus one (FMO) controls (**Supplementary Figure 1C**). Representative example of *n* = 3 biological replicates.

Following confirmation of specificity, the purified CD161 clone 1F2/3A4 was conjugated to Alexa Fluor 568 (AF568) fluorophore for use within the multicolor flow cytometry staining panels. The co-expression of CD161 with NKp46, γδTCR, CD4, and CD8 on PBMCs was investigated through multicolor flow cytometry (**Figure 4**).

Subsets of NK cells were identified through NKp46/CD161 double staining (**Figure 4A**), with the majority of NKp46^+^ cells also expressing CD161. Negligible co-expression of CD161 was observed with CD4 (**Figure 4B**); however, a small population of CD161^Lo^γδTCR^+^ cells was observed (**Figure 4C**). Differential expression of CD161 was shown on CD8^+^ cells (**Figure 4D**), and further multicolor staining demonstrated the CD8^+^CD161^Lo^ cells to be predominantly NKp46^−^, therefore likely to be T cells, and CD8^Lo^CD161^Hi^ to be almost exclusively NKp46^+^, and therefore NK cells.

### 3.5 CD161 expression on NKp46/CD2 NK cell subsets

**Figure 5:**
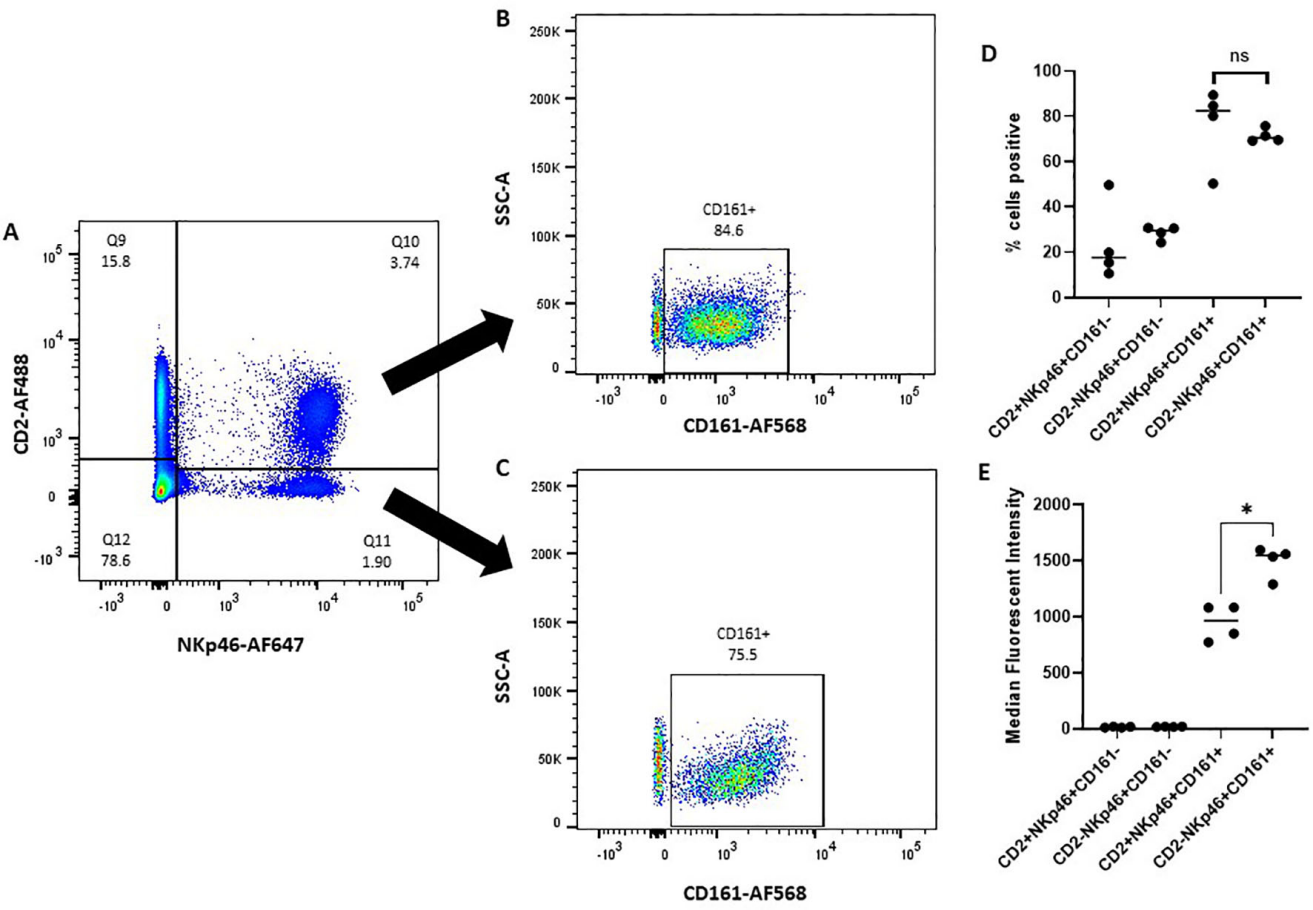
CD161 expression within bovine NKp46/CD2 subsets in bovine peripheral blood. **(A)** CD2^+^NKp46^+^ and CD2^−^NKp46^+^ subsets within bovine peripheral blood mononuclear cells (PBMCs) were defined by flow cytometry. **(B, C)** CD161 expression was defined in each subset. **(D, E)** The percentage **(D)** and the median fluorescent intensity (MFI) **(E)** of CD161-positive cells in the CD2^+^NKp46^+^ and CD2^−^NKp46^+^ subsets were compared using paired *t*-tests (*n* = 4). All plots were gated on single, live cells as described in **Supplementary Figure 1B**, and gates were drawn based on relevant fluorescence minus one (FMO) controls (**Supplementary Figure 1C**). Representative example of *n* = 4 biological replicates.

As previously reported (8), bovine NK cell subsets are commonly defined by the expression of CD2 and NKp46 (**Figure 5A**).

Multi-parametric flow cytometry showed that CD161^+^ and CD161^−^ populations were found within both the CD2^+^NKp46^+^ (**Figure 5B**) and CD2^−^NKp46^+^ subsets (**Figure 5C**). CD161 was expressed by the majority of NK cells in both the CD2^+^NKp46^+^ (average = 82.65%, range = 50.8%–89.5%, *n* = 4) and CD2^−^NKp46^+^ subsets (average = 71.25%, range = 68.8%–75.5%, *n* = 4), and there was no significant difference in the percentage of positive cells between these populations (paired *t*-test) (**Figure 5D**). The median fluorescent intensity (MFI) of the CD161^+^ cells was significantly higher (*p* = 0.023, paired *t*-test) on the CD2^−^NKp46^+^ than the CD2^+^NKp46^+^ subset (**Figure 5E**).

## 4 Discussion

This study developed and characterized a novel mouse anti-bovine CD161 mAb and defined its expression on bovine peripheral blood at baseline levels in calves. Novel subsets of CD161^+^ NK cells, T cells, and γδT cells were described.

Previously, bovine NK cells have been commonly defined using NKp46 expression, often in combination with CD2 (8). The distinct NKp46^+^CD2^+^ and NKp46^+^CD2^−^ subsets carry out differing functions within the bovine immune response, with NKp46^+^CD2^−^ cells predominately producing IFNγ following infection, stimulation, or vaccination (9). Here, we have shown that these subsets can be further separated into CD161^+^ and CD161^−^, with CD161^−^ consistently shown as the minor subset. The function of CD161^+^ NK cells in cattle is yet to be fully defined; however, the possibility that these NK cell subsets may perform differing functions in the immune response to infection, disease, and/or vaccination can now be further explored due to the existence of the novel reagent described here. NKp46^+^CD161^−^ NK cells, although a very small percentage of peripheral blood overall (**Figure 4A**), also warrant further investigation to determine their potential function and role within the immune response to infection, disease, and vaccination. Interestingly, the percentage of cells that express CD161 was comparable between the CD2/NKp46 subsets; however, CD2^−^NKp46^+^CD161^+^ cells were shown to have a significantly higher CD161 MFI than CD2^+^NKp46^+^CD161^+^ cells. This suggests that the CD2^−^ subset, which has been shown to predominantly produce IFNγ in response to stimulation (9), expresses higher levels of CD161. This aligns with data from humans suggesting that CD161 is expressed on a subset of pro-inflammatory NK cells (1).

The expression of CD161 on CD8^+^ T cells and γδTCR cells was also shown. Previous studies of bovine PBMCs showed bovine T cells to have high levels of expression of CD8 (16), whereas NK cells (17) and NKT-like cells (18) expressed lower levels. Therefore, it is likely that the CD161^Hi^CD8^Lo^ population, made up almost exclusively of NKp46^+^, comprises NK or NKT-like cells and that the CD8^Hi^CD161^Lo^ are CD3^+^ cytotoxic T cells. Although in humans the majority of CD8^+^CD161^+^ cells are MAIT cells (19), in the human gut (20) and peripheral blood (21), a subset of CD161^Int^CD8^+^ cells have been described as memory cells. In contrast to the findings in human peripheral blood (3, 22), no CD161^+^CD4^+^ cell subset was observed in cattle. A number of human studies suggest that CD161^+^CD4^+^ subsets are Th17 cells associated with inflammatory and autoimmune disorders (23); therefore, these may not be present in the healthy animals assessed here.

MAIT cells are a population of innate-like T cells that have been linked to immune responses to infections caused by bacteria such as *Mycobacterium* spp. (24). In previous studies, MAIT cells have been defined by the use of an MR1 tetramer, which shows highly conserved reactivity across a number of species (25). Expression of high levels of CD161 has been described as a defining feature of human MAIT cells (26). Intriguingly, we did not observe any CD161^Hi^CD8^+^ cells, which in humans are predominantly MAIT cells. Further studies examining CD161 expression in MAIT cells are therefore required to determine whether its expression differs between cattle and humans.

Further research into the phenotype and function of distinct subsets in bovine peripheral blood is now possible due to the availability of the novel anti-bovine CD161 mAb described here. This could include the measurement of cytotoxic capability through the expression of perforin and granzymes, as well as cytokine production, following stimulation. A specific area of future study would be the potential role of CD161 in the NK cell-mediated protective immunity through BCG vaccination. As the only vaccine currently available to control TB (27), a devastating disease that affects both humans and cattle worldwide, further understanding of the mechanisms involved in the protective response of this vaccine is vital to inform and influence the development of future targeted therapeutics and advances in vaccine development.

## Data Availability

The original contributions presented in the study are included in the article/**Supplementary Material**. Further inquiries can be directed to the corresponding author.

## Supplementary material

Supplementary Figure 1 Example flow cytometry gating strategy and FMO controls. An example gating strategy for the testing of purified monoclonal antibody (mAb) by flow cytometry is shown **(A)**. Debris is excluded by FSC-A v. SSC-A, single cells selected by FSC-H v. FSC-A and viable cells determined by Sytox blue. For multicolor flow cytometry a similar gating strategy is shown **(B)**. Debris is excluded by FSC-A v. SSC-A, single cells selected by FSC-H v. FSC-A, viable cells determined by Zombie NIR and the lymphocyte cell region selected by FSC-A vs. SSC-A. Fluorescent Minus One (FMO) control samples were used to determine gates applied to multi-color stained samples **(C)**.

## References

[1] Kurioka A Cosgrove C Simoni Y Van Wilgenburg B Geremia A Björkander S . CD161 defines a functionally distinct subset of pro-inflammatory natural killer cells. Front Immunol. (2018) 9:486. doi: 10.3389/fimmu.2018.00486. PMID: 29686665 PMC5900032

[2] Montaldo E Vitale C Cottalasso F Conte R Glatzer T Ambrosini P . Human NK cells at early stages of differentiation produce CXCL8 and express CD161 molecule that functions as an activating receptor. Blood. (2012) 119:3987–96. doi: 10.1182/blood-2011-09-379693. PMID: 22403260

[3] Wyrożemski Ł Qiao SW . Immunobiology and conflicting roles of the human CD161 receptor in T cells. Scand J Immunol. (2021) 94:e13090. doi: 10.1111/sji.13090 35611672

[4] Tong B Wang M Liu L Yang X . Immunobiology roles of the human CD161 receptor in T cells. Front Immunol. (2025) 16:1648305. doi: 10.3389/fimmu.2025.1648305. PMID: 40895554 PMC12391111

[5] Van Der Geest KSM Kroesen BJ Horst G Abdulahad WH Brouwer E Boots AMH . Impact of aging on the frequency, phenotype, and function of CD161-expressing T cells. Front Immunol. (2018) 9:752. doi: 10.3389/fimmu.2018.00752. PMID: 29725326 PMC5917671

[6] Walker LJ Kang YH Smith MO Tharmalingham H Ramamurthy N Fleming VM . Human MAIT and CD8αα cells develop from a pool of type-17 precommitted CD8+ T cells. Blood. (2012) 119:422–33. doi: 10.1182/blood-2011-05-353789. PMID: 22086415 PMC3257008

[7] Endsley JJ Endsley MA Estes DM . Bovine natural killer cells acquire cytotoxic/effector activity following activation with IL-12/15 and reduce Mycobacterium bovis BCG in infected macrophages. J Leukoc Biol. (2006) 79:71–9. doi: 10.1189/jlb.0505239. PMID: 16275895

[8] Boysen P Storset AK . Bovine natural killer cells. Vet Immunol Immunopathol. (2009) 130:163–77. doi: 10.1016/j.vetimm.2009.02.017. PMID: 19339058

[9] Boysen P Olsen I Berg I Kulberg S Johansen GM Storset AK . Bovine CD2-/NKp46+ cells are fully functional natural killer cells with a high activation status. BMC Immunol. (2006) 7:10. doi: 10.1186/1471-2172-7-10. PMID: 16643649 PMC1482717

[10] Hao L Klein J Nei M . Heterogeneous but conserved natural killer receptor gene complexes in four major orders of mammals. Proc Natl Acad Sci USA. (2006) 103:3192–7. doi: 10.1073/pnas.0511280103. PMID: 16492762 PMC1413923

[11] Percie du Sert N Hurst V Ahluwalia A Alam S Avey MT Baker M . The ARRIVE guidelines 2.0: Updated guidelines for reporting animal research. PloS Biol. (2020) 18:e3000410. doi: 10.1136/bmjos-2020-100115. PMID: 32663219 PMC7360023

[12] Grevelinger J Bourry O Schmidt S Meurens F Deblanc C Hervet C . Swine influenza A virus infection sets the local immunological landscape in subsequent infection with porcine reproductive and respiratory syndrome virus. Vet Res. (2025) 56:114. doi: 10.1186/s13567-025-01536-6. PMID: 40484956 PMC12147356

[13] Waddell LA Wu Z Sauter KA Hope JC Hume DA . A novel monoclonal antibody against porcine macrophage colony-stimulating factor (CSF1) detects expression on the cell surface of macrophages. Vet Immunol Immunopathol. (2023) 266:110681. doi: 10.1016/j.vetimm.2023.110681. PMID: 37992576

[14] Khalid H Coad M Dry I McGuinnes C Waddell LA Hope JC . Development and characterization of monoclonal antibodies specific for bovine IP-10. Vet Res. (2025) 56:169. doi: 10.21203/rs.3.rs-6304513/v1. PMID: 40814003 PMC12351912

[15] Hanton AJ Waddell LA Hope JC Gray M Wu Z . Bovine NK subsets in the afferent lymph and lymph nodes have distinct expression of naïve and activation-associated cell surface expressed molecules, and are differentially stimulated by BCG vaccination. Vet Immunol Immunopathol. (2023) 266:110682. doi: 10.1016/j.vetimm.2023.110682. PMID: 38000215

[16] Sopp P Howard CJ . Cross-reactivity of monoclonal antibodies to defined human leucocyte differentiation antigens with bovine cells. Vet Immunol Immunopathol. (1997) 56:11–25. doi: 10.1016/s0165-2427(96)05731-5. PMID: 9220577

[17] Storset AK Kulberg S Berg I Boysen P Hope JC Dissen E . NKp46 defines a subset of bovine leukocytes with natural killer cell characteristics. Eur J Immunol. (2004) 34:669–76. doi: 10.1002/eji.200324504. PMID: 14991596

[18] Connelley TK Longhi C Burrells A Degnan K Hope J Allan AJ . NKp46+ CD3+ cells: a novel nonconventional T cell subset in cattle exhibiting both NK cell and T cell features. J Immunol. (2014) 192:3868–80. doi: 10.4049/jimmunol.1302464. PMID: 24639352 PMC3990274

[19] Ussher JE Bilton M Attwod E Shadwell J Richardson R De Lara C . CD161++ CD8+ T cells, including the MAIT cell subset, are specifically activated by IL-12+IL-18 in a TCR-independent manner. Eur J Immunol. (2014) 44:195–203. doi: 10.1002/eji.201343509 24019201 PMC3947164

[20] Fergusson JR Hühn MH Swadling L Walker LJ Kurioka A Llibre A . CD161(int)CD8+ T cells: a novel population of highly functional, memory CD8+ T cells enriched within the gut. Mucosal Immunol. (2016) 9:401–13. doi: 10.1038/mi.2015.69. PMID: 26220166 PMC4732939

[21] Konduri V Oyewole-Said D Vazquez-Perez J Weldon SA Halpert MM Levitt JM . CD8(+)CD161(+) T-Cells: Cytotoxic memory cells with high therapeutic potential. Front Immunol. (2020) 11:613204. doi: 10.3389/fimmu.2020.613204. PMID: 33597948 PMC7882609

[22] Fergusson JR Smith KE Fleming VM Rajoriya N Newell EW Simmons R . CD161 defines a transcriptional and functional phenotype across distinct human T cell lineages. Cell Rep. (2014) 9:1075–88. doi: 10.1016/j.celrep.2014.09.045. PMID: 25437561 PMC4250839

[23] Belpaire A Van Geel N Speeckaert R . From IL-17 to IFN-γ in inflammatory skin disorders: Is transdifferentiation a potential treatment target? Front Immunol. (2022) 13:932265. doi: 10.3389/fimmu.2022.932265. PMID: 35967358 PMC9367984

[24] Edmans MD Connelley TK Jayaraman S Vrettou C Vordermeier M Mak JYW . Identification and phenotype of MAIT cells in cattle and their response to bacterial infections. Front Immunol. (2021) 12:627173. doi: 10.3389/fimmu.2021.627173. PMID: 33777010 PMC7991102

[25] Edmans MD Connelley TK Morgan S Pediongco TJ Jayaraman S Juno JA . MAIT cell-MR1 reactivity is highly conserved across multiple divergent species. J Biol Chem. (2024) 300:107338. doi: 10.1016/j.jbc.2024.107338. PMID: 38705391 PMC11190491

[26] Dusseaux M Martin E Serriari N Péguillet I Premel V Louis D . Human MAIT cells are xenobiotic-resistant, tissue-targeted, CD161hi IL-17-secreting T cells. Blood. (2011) 117:1250–9. doi: 10.1182/blood-2010-08-303339. PMID: 21084709

[27] Tagliabue A Boraschi D Leite LCC Kaufmann SHE . 100 years of BCG immunization: Past, present, and future. Vaccines (Basel). (2022) 10(10):1743. doi: 10.3390/vaccines10101743. PMID: 36298608 PMC9610200

