# Supplementary figures and images for: Bovine natural killer cell subsets can be defined by differential expression of CD161 (KLRB1)
Source: Front Immunol. 2026 Jun 3;17:1843038. doi: 10.3389/fimmu.2026.1843038 (PMC13271940; doi:10.3389/fimmu.2026.1843038)

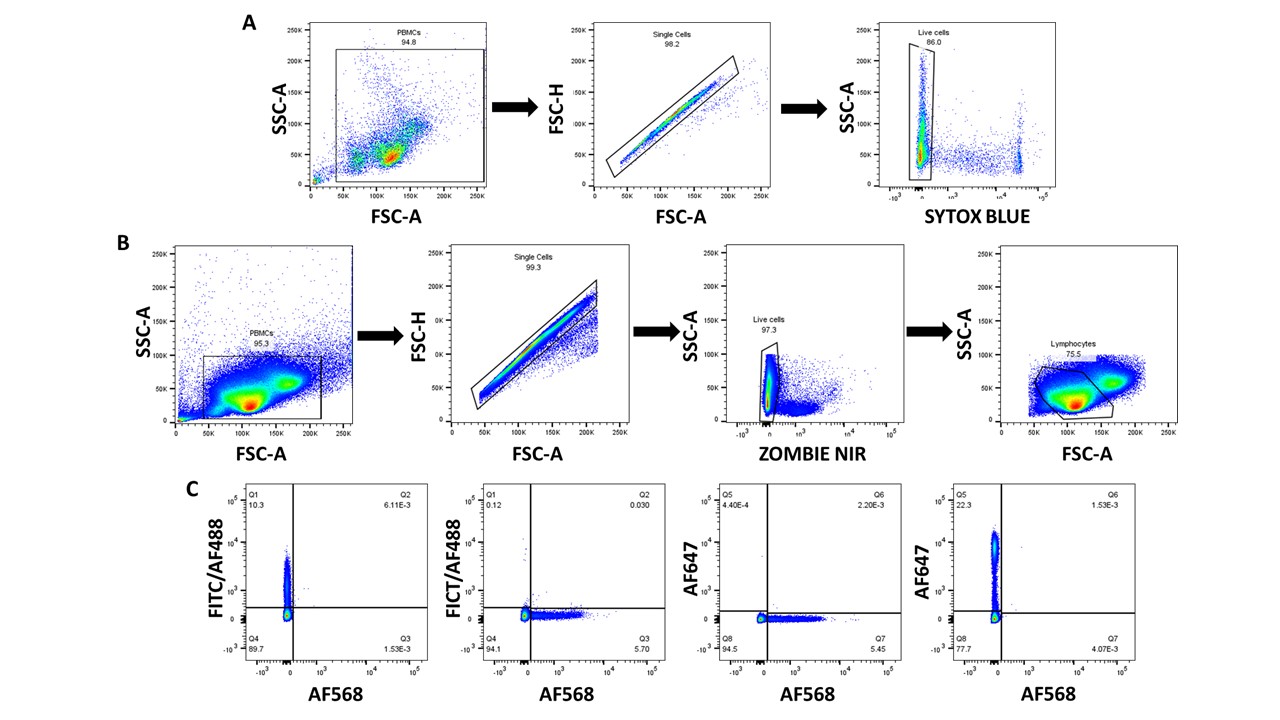

Supplement: Supplementary Figure 1 — Example flow cytometry gating strategy and FMO controls. An example gating strategy for the testing of purified monoclonal antibody (mAb) by flow cytometry is shown (A). Debris is excluded by FSC-A v. SSC-A, single cells selected by FSC-H v. FSC-A and viable cells determined by Sytox blue. For multicolor flow cytometry a similar gating strategy is shown (B). Debris is excluded by FSC-A v. SSC-A, single cells selected by FSC-H v. FSC-A, viable cells determined by Zombie NIR and the lymphocyte cell region selected by FSC-A vs. SSC-A. Fluorescent Minus One (FMO) control samples were used to determine gates applied to multi-color stained samples (C). [file Image1.jpeg]
